# Editorial: Biomolecular modifications in endocrine-related cancers

**DOI:** 10.3389/fendo.2023.1133629

**Published:** 2023-01-13

**Authors:** Xianquan Zhan, Junwen Su, Lamei Yang

**Affiliations:** Medical Science and Technology Innovation Center, Shandong Key Laboratory of Radiation Oncology, Shandong Cancer Hospital and Institute, Shandong First Medical University & Shandong Academy of Medical Sciences, Jinan, Shandong, China

**Keywords:** endocrine-related cancers, DNA modifications, post-transcriptional modifications, post-translational modifications, genomics, transcriptomics, proteomics, proteoformics

The central dogma of genetics explains the sequence of information flow among three levels of biomolecules: from DNA, to RNA, to proteins (1). Modifications occurring in these biomolecules are key molecular events that regulate their structures and functions, and even the entire biological processes and systems in which they are involved (2). Biomolecular modifications are recognized as playing extensive roles in various pathophysiological conditions, including diabetics, metabolic disease, neurodegenerative disease, inflammatory disease, and cancer. Biomolecular modification is also a highly important factor in biomolecule diversity (3). It is estimated that at least 10 types of modifications, such as hydroxymethylation and cytosine methylation, occur in DNA, while at least 20 post-translational modifications (PTMs), such as acetylation, ubiquitination, and phosphorylation, occur in DNA-binding protein histones, which regulate the structures and functions of DNA; at least 170 post-transcriptional modifications, such as N1- and N6-methyladenosines (m1A, m6A, m6Am), 5-hydroxymethylcytosine (hm5C), 2′-O-methylation (Nm), 3- and 5-methylcytosines (m3C, m5C), and pseudouridine (Ψ), occur in RNA and thereby regulate the structures and functions of RNA; and 400-600 PTMs, such as glycosylation, acetylation, methylation, phosphorylation, ubiquitylation, SUMOylation, nitration, sulfation, hydroxylation, deamidation, prenylation, nitrosylation, succinylation, palmitoylation, and myristoylation, occur in proteins and thereby regulate the structures and functions of proteins (**Figure 1**) (1–3). Each biomolecular modification has its own characteristics and associated research methodologies. The development of various omics, including genomics, transcriptomics, proteomics, proteoformics, and bioinformatics, has been a significant driver of the large-scale analysis of biomolecular modifications to determine the modified sites and levels of modification, and to further elucidate the molecular mechanisms and biofunctions mediated by biomolecular modifications (3). Moreover, synergistic and antagonistic interactions between different biomolecular modifications can complicate the biological effects of a biomolecule to a remarkable extent. Up to this point, the number of biomolecular modification studies undertaken in the area of medical and life sciences has been far from adequate (3). It is time to put further effort into the broad and deep study of biomolecular modifications.

**Figure 1:**
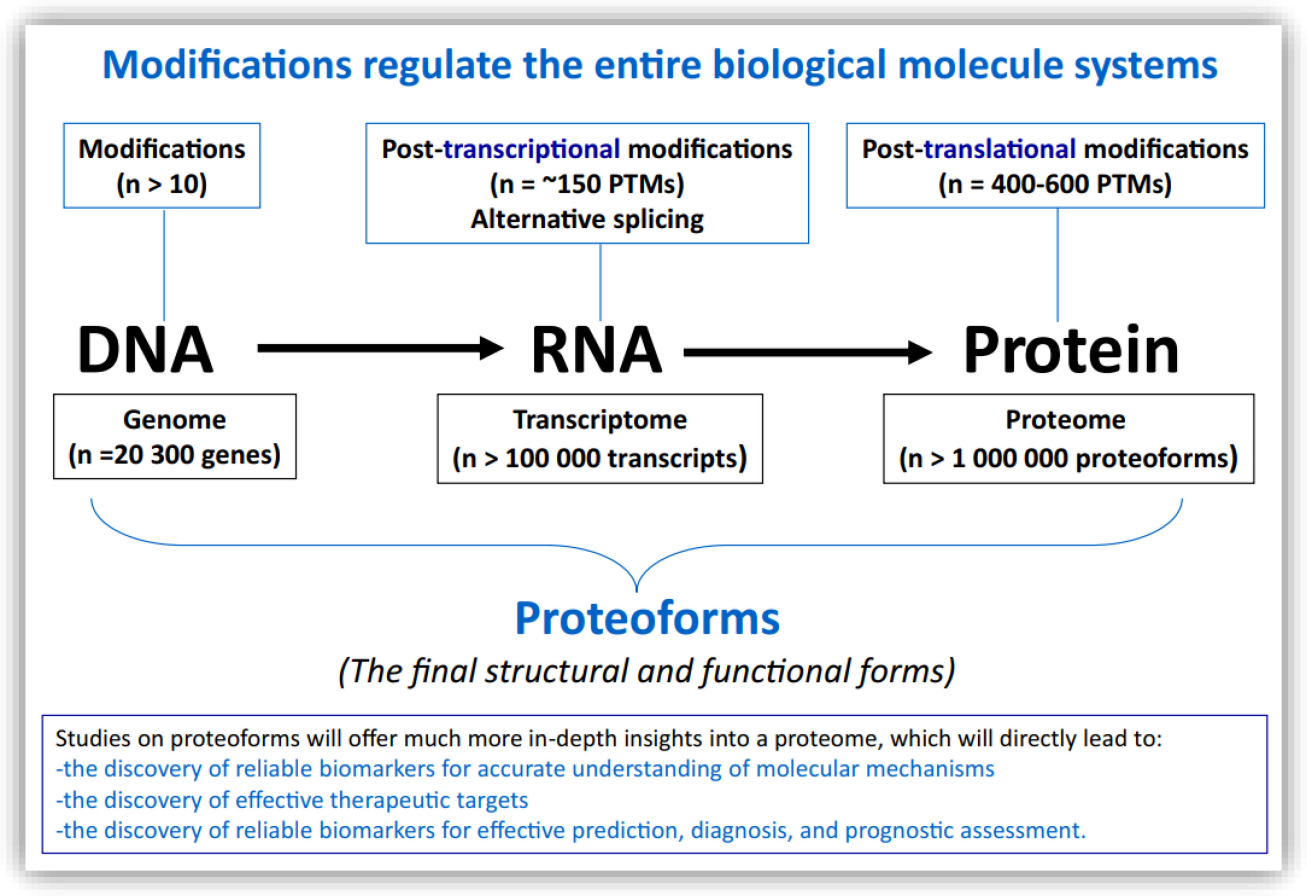
The relationships between biomolecular modifications in DNA, RNA, and protein, and the proteoforms that constitute the end-point structural and functional forms of a gene or protein. Constructed from Li, Desiderio, and Zhan (1) with copyright permission from Wiley, and from Zhan, Long, and Lu (3) with copyright permission from Elsevier. Reproduced from Zhan X et al. (2) with copyright permission from Springer Nature.

Endocrine-related cancers, such as ovarian cancer, pituitary adenoma, thyroid cancer, lung cancer, and breast cancer, are important endocrine system disorders and each involves a series of biomolecular modifications at the levels of DNA, RNA, and proteins. These biomolecular modifications are crucial factors contributing to the formation and development of endocrine-related cancers, in that they precisely regulate the molecular mechanisms underlying tumor-related pathophysiological processes, which in turn are a source of effective therapeutic targets and drugs, and reliable biomarkers of endocrine-related cancers (1–3). The present Research Topic includes articles discussing the presentation, functional roles, and mechanisms of various biomolecular modifications in endocrine-related cancers; this will pave the way for the systematic study of biomolecular modifications at the levels of DNA, RNA, and proteins in endocrine-related cancers.

A total of 11 articles are presented in this issue. (i) The first article addresses quantitative acetylomic alterations and the signaling pathway changes mediated by these alterations in human nonfunctional pituitary adenomas (Wen et al.). The authors identify 296 acetylated proteins with 517 acetylation sites, observing that most of these exhibit decreased acetylation levels in pituitary adenomas and play a role in multiple biological processes, including oxidative stress, cell adhesion, translation, and metabolism. Additionally, this study indicates that acetylation-mediated metabolic reprogramming contributes to invasive behaviors of the pituitary adenoma. (ii) The second article addresses protein pupylation; the authors construct the first pupylation prediction model for accurate prediction of pupylation sites and pupylated proteins, which is of great significance for the study of basic biological processes and the development of pupylation-related drugs (Qiu et al.). (iii) The third article addresses the use of quantitative ubiquitinomics to reveal abnormal ubiquitinated ATP7A involved in down-regulation of ACTH secretion in silent corticotroph adenomas (Zhao et al.), and clearly demonstrates the effect of ubiquitylation on ACTH secretion in silent corticotroph adenomas. (iv) The fourth article addresses the roles and potential mechanisms of RSUME proteoforms, which are a set of small RWD domain-containing proteins that play a role in enhancing SUMO conjugation in tumorigenesis (Fuertes et al.). (v) The fifth article addresses the roles and therapeutic implications of epigenetic modifications, such as histone modifications, DNA methylation, and non-coding RNA regulation, in ovarian cancer; the authors also discuss the relationships of epigenetic modifications with multidrug resistance, the tumor microenvironment, and the immune response in tumorigenesis (Wang et al.). (vi) The sixth article addresses the concept of glycomics and associated methodologies, reviewing glycomics-derived biomarkers and therapeutic targets in cancers within the framework of 3P medicine (Guo et al.). (vii) The seventh article addresses ubiquitination-mediated signaling pathway changes in human lung squamous cell carcinomas (LSCC). The authors identify 627 ubiquitinated proteins (UPs) with 1209 ubiquitination sites, thereby providing an initial view of the landscape of UPs and molecular networks for human LSCC tissue (Zhan et al.). (viii) The eighth article addresses concepts and methodologies relating to protein acetylation, which is dynamically regulated by histone deacetylases (HDACs) and histone acetyltransferases (HATs) in homeostasis; the authors review insights into acetylation-based mechanisms in carcinogenesis and targeted drug discovery in cancers (Yang et al.). (ix) The ninth article addresses eight types of RNA modification (m3C, ac4C, m7G, m5C, m6A, m1A, m6Am, and Ψ) and the corresponding RNA-modification regulatory genes (RRGs; n = 59). The authors analyze the variation in expression and the clinical relevance of these 59 RRGs in ovarian cancers, thereby constructing a differentially expressed RRG signature model for ovarian cancer consisting of four RRGs (ALYREF, ZC3H13, WTAP, and METTL1), which can be used as an independent prognostic model to classify ovarian cancer patients into high- and low-risk groups (Zheng et al.). (x) The tenth article addresses the biomolecular modifications occurring in dedifferentiated thyroid cancer, such as ubiquitination, phosphorylation, acetylation, glycosylation, and DNA methylation, and identifies new targets for radiological imaging and therapy, promising an era of precise diagnosis of and treatment for dedifferentiated thyroid cancer (Li et al.). (xi) Finally, the eleventh article presents clear evidence that PKC-mediated phosphorylation and activation of the MEK/ERK pathway is the mechanism underlying acquired trastuzumab resistance in HER2-positive breast cancer. This represents a typical application of targeted phosphorylation profiling in a given signaling pathway in the study of HER2-positive breast cancer (Scerri et al.).

As the above summary clearly demonstrates, the present issue covers the spectrum of biomolecular modifications at three different levels (those of DNA, RNA, and proteins), including histone modification, DNA methylation, and non-coding RNA regulation at the DNA level for ovarian cancer (Wang et al.) or dedifferentiated thyroid cancer (Li et al.); post-transcriptional modifications, such as m1A, m6A, m6Am, m5C, m7G, ac4C, m3C, and Ψ, at the RNA level for ovarian cancer (Zheng et al.); and post-translational modifications, such as acetylation (Wen et al.; Yang et al.), pupylation (Qiu et al.), ubiquitination (Zhao et al.; Zhan et al.), glycosylation (Guo et al.), phosphorylation (Scerri et al.), and RSUME proteoforms (Fuertes et al.), at the protein level for different forms of carcinogenesis. The aforementioned studies examining these molecular modifications contribute to the development of in-depth insight into the molecular mechanisms of cancers, such as the mediation of signaling pathway alterations by biomolecular alterations (Wen et al.;Zhan et al.; Scerri et al), the identification of effective cancer biomarkers (Yang et al.; Guo et al.; Wen et al.; Zhan et al), and the discovery of effective therapeutic targets and drugs in cancer treatment (Yang et al.; Guo et al.). Omics represent effective approaches in the study of biomolecular modification for the large-scale identification of modification sites and quantification of modification levels (Wen et al.; Zhan et al.; Zhao et al.). Moreover, data on biomolecular modifications, in combination with other types of omics data, have the potential for clinical applications. For example, an understanding of RNA modifications can be usefully combined with transcriptomics data (Zheng et al.); acetylation can be exploited in combination with transcriptomics to resolve invasive behaviors in pituitary adenomas (Wen et al.); and protein post-translational modifications can be used in combination with radiomics (Li et al.). For these reasons, biomolecular modifications are of crucial importance, but have not been sufficiently investigated in the field of cancers, where they are involved in every aspect of predictive, preventive, and personalized medicine (3P medicine). Biomolecular modifications are an important factor in diversity in proteoforms, each of which is defined by its amino acid sequence + PTMs + spatial conformation + cofactors + binding partners + localization + a function (4, 6–8). Proteoforms are the basic units of a proteome, and represent the final structural and functional forms of a gene or a protein. We recommend that the study of “proteoformics” be strengthened in order to clarify the precise structural and functional alterations of a given molecule; this will enable the discovery of effective biomarkers for an accurate understanding of molecular mechanisms in cancer, the identification of reliable therapeutic targets and drugs, and the precise prediction, diagnosis, and assessment of prognosis in any given cancer (9–12).

In summary, significant advances have been achieved through biomolecular modification studies in the domain of endocrine-related cancers. The present issue collects various important articles on the topics of biomolecular modifications in DNA, DNA-binding protein histones, RNA, and proteins in endocrine-related cancers, and on the combination of various biomolecular modifications with other omics-related data in the identification of cancer biomarkers. However, it must be acknowledged that this special issue tackles only a very small fraction of the biomolecular modifications that are relevant in endocrine-related cancers. This Research Topic serves as a catalyst to stimulate and encourage researchers to conduct further biomolecular modification studies, which will result in important scientific developments in research and clinical practice in the domain of endocrine-related cancers. Future special issues will collect further multiomics-based studies of biomolecular modifications involving the large-scale use of clinical information in basic, translational, and clinical practice research in endocrine-related cancers. We will emphasize studies examining synergistic and antagonistic interactions among biomolecular modifications in a given biomolecule and their biological roles in various endocrine-related cancers. We strongly believe that biomolecular modification-based proteoformics studies represent a brighter future for the treatment of endocrine-related cancers within the framework of predictive, preventive, and personalized medicine (PPPM; 3P medicine) and precision medicine.

## Author contributions

XZ conceived the concept; collated and analyzed the literature; planned, wrote, and critically revised the manuscript; and was responsible for financial support for this and related work. JS and LY participated in the collation of the literature and in part of the writing. All authors approved the final manuscript.
